# Primary tubercular abscess of the breast – an unusual entity

**Published:** 2012-03-05

**Authors:** R Gupta, RP Singal, A Gupta, S Singal, SR Shahi, R Singal

**Affiliations:** Department of Surgery, Adesh Institute of Medical Science and Research, Bathinda, Punjab, India; Department of Orthopedics, Adesh Institute of Medical Science and Research, Bathinda, Punjab, India; Department of Anatomy, Adesh Institute of Medical Science and Research, Bathinda, Punjab, India; Dept of Gynecology, Multispecialty Hospital, C/o Dr Kundan Lal Hospital, Ahmedgarh, Punjab, India; Dept of Medicine and Ayurvedic, C/o Dr Kundan Lal Hospital, Multispecialty Hospital, Ahmedgarh, Punjab, India.; Dept of Surgery, Multispecialty Hospital, C/o Dr Kundan Lal Hospital, Ahmedgarh, Punjab, India

**Keywords:** Extra – pulmonary, lump, tuberculosis, management

## Abstract

Primary breast tuberculosis manifested as abscess is a rare entity. We are reporting a case of primary breast tuberculosis, which presented as breast abscess. Abscess was drained and tissue sent for histopathology. To our surprise, diagnosis came as breast tuberculosis. Aspiration cytology was not done, as it is not a routine test for abscess cases. Patient was put on anti- tubercular drugs. In the follow-up of 6 months, she was asymptomatic and advised to continue medicine.

## Introduction

Breast tuberculosis (TB) is a rare form of extra pulmonary TB which was first described by Sir Astley Cooper [**1**]. Although worldwide, over one billion people suffer from TB, mammary tuberculosis is an extremely rare condition. Its primary form is even more infrequent. The incidence of isolated TB of the breast ranges from 0.10% to 0.52% is scarcely reported even in countries with a high incidence of tuberculosis infection [**2**]. This is explained by a noticeable resistance of the mammary tissue to the mycobacterium tuberculosis [**3**]. 

## Case report

A 42-year-old female reported with pain in the right breast for two months, along with off and on fever. She started feeling heaviness in her right breast 20 days before. There were no other complaints. Patient took treatment from the local practioner but there was no relief. 

On examination of the right breast, local temperature was raised and a tender lump of about 4x6 cm was felt in the upper outer quadrant. Lump was firm in consistency and non-mobile. Signs of inflammation were present. Provisional diagnosis was made as breast abscess.

**Fig. 1:**
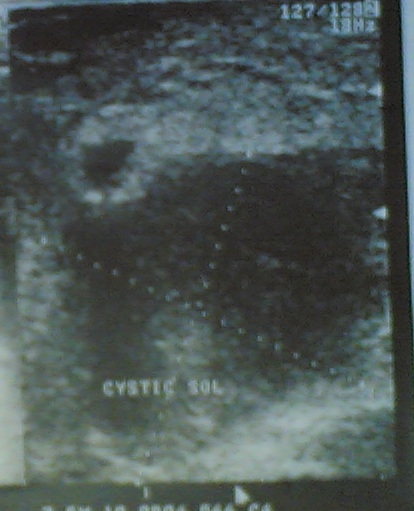
Ultrasonography of breast showed hypoechoic lesion with multiple internal echoes

Total leukocyte counts and erythrocyte sedimentation rate was raised. Rests of blood tests were within normal limits including chest X-ray. Ultrasonography of the breast revealed a large homogeneous capacity in right breast with area of asymmetrical density (**Fig. 1**).

**Fig. 2:**
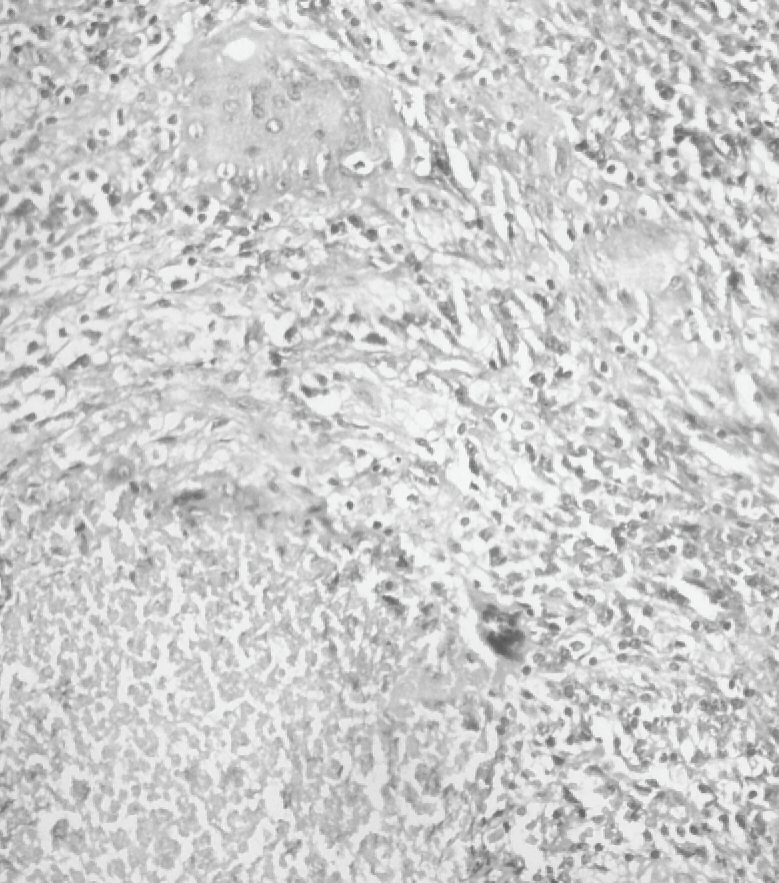
Photomicrograph showing caseating granuloma in the breast (H & E, X – 200)

On needle aspiration, thick pus came out. Incision given and thick pus was drained out of about 100 ml. Tissue and pus was sent for histopathology and, to our surprise, the diagnosis came as breast tuberculosis (**Fig. 2**). 

The patient was put on anti-tubercular drugs (rifampicin 600 mg, isoniazid 300 mg, pyrazinamide 1500 mg and ethambutol 1000 mg per day) for 2 months and continued with the addition of rifampicin and isoniazid therapy for 4 additional months. In the follow-up period of 6 months, the patient recovered very well and was advised to continue the treatment.

## Discussion

Isolated TB breast is an uncommon condition, even in developing countries where pulmonary and other forms of extrapulmonary manifestations of TB are endemic. The incidence of isolated TB of the breast remains low, ranging from 0.10% to 0.52%. In the high tubercular endemic countries like India, the incidence represents 3 to 4.5% of the mammary pathologies [**4**]. In the Western countries, with a lower tubercular incidence, it represents less than 0.1% of the mammary lesions examined via histology [**3,5**]. TB of the breast usually affects women aged between 20 and 50 years. Breast involvement can be either primary without any extra-mammary focus, or secondary to pulmonary tuberculosis. The primary form of the disease is rare [**6**].

Breast tissue, along with skeletal muscle and spleen, appears to be relatively resistant to tuberculous infection [**7**]. The commonest location of the lump in breast is the central or upper outer quadrant of the breast [**8**]. The mass may be fluctuant and is usually covered with indurated tissue. It is usually fixed to the skin and fistulization is not uncommon. Nipple and skin retraction can also occur, but breast discharge and pain are not common [**9**]. 

It may be classified into three types, namely: nodular, disseminated and sclerosing varieties. McKeown and Wilkinson classified tuberculosis of the breast into five different types: the three stated above and acute miliary tuberculosis mastitis and tuberculosis mastitis obliterans [**10**]. The nodular form is the most common variety and is characterized by a well defined, painless, slow growing caseous lesion in the breast. Involvement of overlying tissue is usually late and it is at this point that the mass becomes painful. As in our case, the patient presented with breast abscess, and, on histology diagnosis, it came as tuberculosis, which is very rare. Ultrasound is useful for characterising the ill-defined densities shown on mammography, by excluding solid masses, but the findings of a hypoechoic lesion with heterogeneous internal echoes and irregular borders are not specific [**2**]. 

Microbiological and histological examinations remain the gold standard for the diagnosis of this uncommon disease. Early diagnosis is difficult, as the characteristic sinuses appear late in the course of the disease. Tuberculosis of the breast is often diagnosed as pyogenic abscess in young women and, in the elderly as carcinoma. In our case breast abscess was diagnosed after the drainage and histopathological examination. The treatment of tuberculosis mastitis is best achieved by conservative surgery and anti-tuberculosis chemotherapy.

## Conclusion

The diagnosis must be considered in young patients presenting with a palpable lump, especially if they are lactating. A histological examination is required for confirmation. We concluded that if patient presented with an abscess, we should also go for aspiration cytology keeping in mind that the treatment will be easy. It is always a must to do tissue biopsy when the clinical impression is merely breast abscess. 
